# Le kyste hydatique du foie: à propos d'un cas inhabituel

**DOI:** 10.11604/pamj.2015.20.295.6577

**Published:** 2015-03-26

**Authors:** Mahmoudi Ammar, Abdelaziz Hamdi

**Affiliations:** 1 Service de Chirurgie Générale et Digestive, CHU Fattouma Bourguiba de Monastir, Tunisie

**Keywords:** Hydatidose, foie, chirurgie, kyste hydatique du foie, Hydatidosis, liver, surgery, hydatid cyst of the liver

## Image en medicine

**Figure 1:**
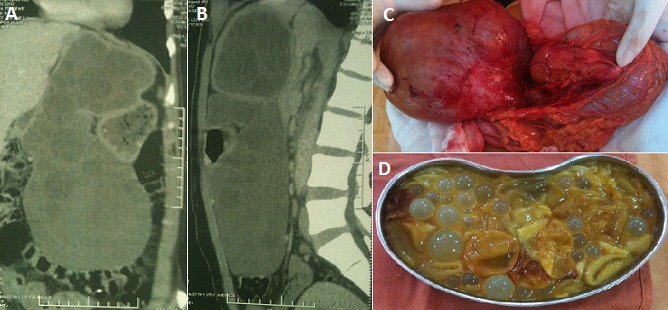
A) scanner abdominal en coupe frontale: énorme lésion kystique multiloculaire prenant naissance à partir du foie gauche et refoulant l'estomac en avant, le pancréas en arrière et le colon transverse vers le bas; B) scanner abdominal en coupe sagittale: énorme lésion kystique multiloculaire prenant naissance à partir du foie gauche et refoulant l'estomac en avant, le pancréas en arrière et le colon transverse vers le bas au-dessous du promontoire; C) vue opératoire montrant le kyste hydatique tenu par la main droite de l'opérateur, l'estomac en arrière de la lésion et le colon transverse tenu par la main gauche; D) contenu du kyste hydatique multi-vésiculaire et bilieux

Le kyste hydatique du foie (KHF) est un parasite de développement lent qui génère des lésions anatomopathologiques variées, responsables de tableaux cliniques divers et polymorphes. Le but du traitement est d’éliminer le parasite, et de régler le problème de la cavité résiduelle et des éventuelles complications associées. Nous rapportons le cas d'un KHF de taille et de topographie inhabituelles puisqu'il prend naissance à partir du foie gauche et plonge le long du petit épiploon pour passer en rétro-gastrique et pré-pancréatique et refoulant très bas le colon transverse. Patiente agée de 34 ans, aux antécédents familiaux d'hydatidose, présentant depuis deux mois une douleur de l'hypocondre droit à type de pesanteur. Il existait une masse palpable occupant l'hypocondre droit, l’épigastre et le flanc gauche. Le scanner abdominal a montré une volumineuse formation kystique multiloculaire faisant 20*15*10 cm étendue du foie gauche jusqu'au flanc gauche évoquant un volumineux KHF. La sérologie hydatique était positive. La patiente a été opérée par une sous-costale droite élargie à gauche. Le KHF était volumineux et occupait la totalité du foie gauche qui est atrophié. Le kyste était ouvert en rétro et sous gastrique pour évacuer son contenu qui est multi-vésiculaire et bilieux. Il a été réalisé une résection du dôme saillant, une fermeture de deux fistulettes kysto-biliaires (objectivées par la cholangiographie per-opératoire et l’épreuve au bleu de méthylène), une épiplooplastie et un drainage de la cavité résiduelle. Les suites opératoires immédiates et lointaines étaient simples en particulier pas de récidive hydatique avec un recul d'un an. Figure 1

